# Characterization of Solar Radiation-Induced Degradation Products of the Plant Sunscreen Sinapoyl Malate

**DOI:** 10.1021/acsagscitech.2c00279

**Published:** 2023-01-19

**Authors:** Matthias J. A. Vink, John J. Schermer, Jonathan Martens, Wybren Jan Buma, Giel Berden, Jos Oomens

**Affiliations:** † Institute for Molecules and Materials, FELIX Laboratory, Radboud University, Toernooiveld 7, 6525 ED Nijmegen, The Netherlands; ‡ Institute for Molecules and Materials, Radboud University, Heyendaalseweg 135, 6525 AJ Nijmegen, The Netherlands; § van’t Hoff Institute for Molecular Sciences, University of Amsterdam, Science Park 904, 1098 XH Amsterdam, The Netherlands

**Keywords:** photomolecular heater, infrared ion spectroscopy, liquid chromatography, mass spectrometry, sinapoyl malate

## Abstract

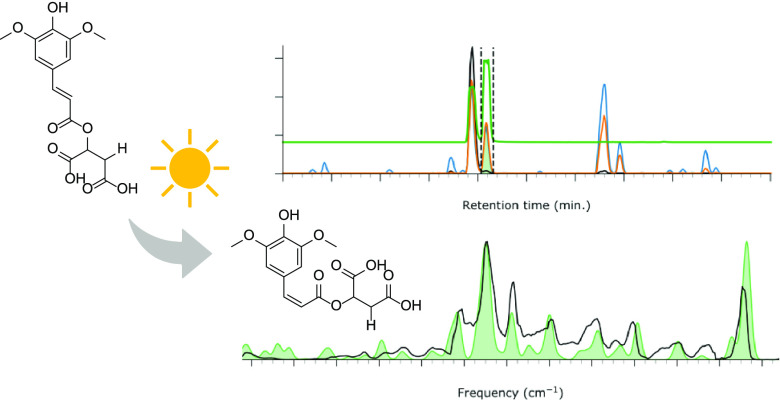

Agricultural activities at lower temperatures lead to lower yields due to reduced plant growth. Applying photomolecular heater agrochemicals could boost yields under these conditions, but UV-induced degradation of these compounds needs to be assessed. In this study, we employ liquid chromatography–mass spectrometry (LC–MS) coupled with infrared ion spectroscopy (IRIS) to detect and identify the degradation products generated upon simulated solar irradiation of sinapoyl malate, a proposed photomolecular heater/UV filter compound. All major irradiation-induced degradation products are identified in terms of their full molecular structure by comparing the IRIS spectra obtained after LC fractionation and mass isolation with reference IR spectra obtained from quantum-chemical calculations. In cases where physical standards are available, a direct experimental-to-experimental comparison is possible for definitive structure identification. We find that the major degradation products originate from *trans*-to-*cis* isomerization, ester cleavage, and esterification reactions of sinapoyl malate. Preliminary in silico toxicity investigations using the VEGAHUB platform suggest no significant concerns for these degradation products’ human and environmental safety. The identification workflow presented here can analogously be applied to break down products from other agrochemical compounds. As the method records IR spectra with the sensitivity of LC–MS, application to agricultural samples, e.g., from field trials, is foreseen.

## Introduction

Innovative and better agrochemical compounds are necessary in a world with a growing population and decreasing quantity and quality of arable land,^1−3^ where food security will inevitably become a significant challenge.^4−6^ Agricultural activities in regions with lower temperatures suffer from limited plant growth and crop yields and necessitate using energy-consuming greenhouses.^7,8^ Recently, research projects have been initiated to investigate the photomolecular heating ability of nature-inspired compounds,^9−16^ which employ a part of the solar radiation that is not used for photosynthesis to raise crop temperature. A temperature increase of only a few degrees can significantly increase crop yields.^8,17^

**Figure 1:**
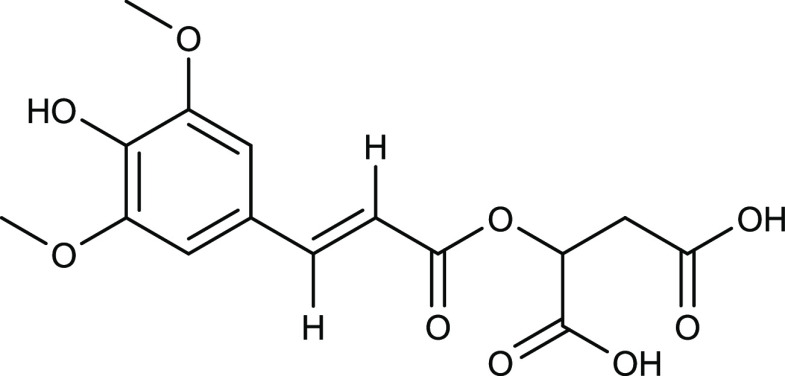
Chemical structure of SM.

One of the candidate compounds, inspired by Nature, is sinapoyl malate (SM), depicted in Figure 1. Sinapic acid derivatives are produced and observed to accumulate in relatively high concentrations in the epidermal layer of leaves and have a photoprotective role.^8,18−23^ Their spatial distribution allows these compounds to absorb harmful radiation before it can cause adverse effects in the underlying tissues, which has generated interest in the scientific community for utilization as environmentally friendly alternative UV filters.^14,18−23^ Efficient UV filters are able to absorb the UV-photon energy into low-lying electronically excited singlet states and efficiently dissipate this energy by internal conversion to the electronic ground state.^24,25^ Various reviews exploring the mechanistic details of these processes by UV laser spectroscopy have recently been published.^24,25^ Facile excited-state cis-trans isomerization drives the fast internal conversion, effectively inducing UV-to-heat conversion pathways. Internal conversion to the ground electronic state in SM is particularly efficient due to the absence of accessible long-lived electronically excited states, making this molecule a particularly effective UV filter designed by Nature.^13,14,23^ It has been proposed to functionalize sinapic acid derivatives to improve their natural ability to filter UV frequencies, producing efficient photomolecular heaters.^13,14,23^

An important issue that needs to be addressed for these chemical-based solutions to agricultural problems is the degradation of the proposed compounds, which increases the chemical complexity of the agrochemical formulation applied to the crop. The profile of degradation products must be characterized to assess their impact on environmental and human safety in regulatory studies.^26−32^ Within the analytical chemistry toolbox, a variety of methods is available to identify the chemical structure of degradation products, including (tandem) mass spectrometry.^33,34^ A major deficiency of MS-based methods is that precise molecular structure characterization necessarily relies on MS/MS libraries or the availability of external standards.^35,36^ De novo structural identification, independent of databases or physical standards, is possible through nuclear magnetic resonance (NMR) spectroscopy, but limitations in sensitivity and/or selectivity and the elaborate purification, therefore, severely limit NMR-based identification workflows.^33,37^

Infrared (IR) spectroscopy provides a unique molecular fingerprint in the form of the normal mode vibrational frequencies and their absorption intensities. Such a fingerprint can differentiate and identify molecular structures but cannot separate the signals resulting from individual constituents in a complex mixture. However, when IR spectroscopy is integrated with mass spectrometry (MS), vibrational spectra can be obtained for mass-selected species from the mixture, taking advantage of the selectivity and sensitivity of liquid chromatography-mass spectrometry (LC–MS) and the structural diagnostics of IR spectroscopy.^38−40^

Here, we record vibrational spectra and derive molecular structures for retention-time and molecular-weight selected ions from UV-exposed sinapoyl malate samples, employing infrared ion spectroscopy (IRIS) implemented on a quadrupole ion trap (QIT) MS platform. The free-electron laser FELIX supplies intense and widely tunable IR radiation for IRIS experiments.^40,41^ This approach allows us to differentiate isomeric UV photoproducts, which is of crucial importance as the photomolecular heating ability of SM is driven by cis-trans isomerization.

The ability of modern quantum-chemical calculations to predict IR spectra with high fidelity enables us to quickly narrow down the list of candidate molecular structures for the photoproducts and achieve tentative but reasonable structural assignments. Implementing IRIS spectroscopy with quantum-chemical computational workflows facilitates a reference standard-free approach to characterize unknown (bio-)transformation products in complex mixture samples.^38,39,42−45^ Although this study focuses on SM, the presented workflow is generically applicable, and a proof-of-concept study on other agrochemical compounds has recently been published.^46^

## Chemicals and Materials

SM was synthesized by AgroParisTech (Paris, France) following a procedure reported previously.^47^ Methanol (MeOH), water, sinapic acid ≥99%, and formic acid (FA) were obtained from Merck (Darmstadt, Germany). All solvents were of LC–MS grade.

## Methods

### Sample Irradiation

SM was dissolved in a mixture of MeOH and water (80/20; v/v%) to a concentration of 10 mM as it does not adequately dissolve in water alone. In addition, the solubility of the byproducts derived from SM was assumed to be higher in MeOH.

For sample irradiation, a custom tubular cuvette with a quartz lid filled for about 70% with SM solution was used in a temperature-controlled sample holder positioned under an ABET Technologies Sun 2000 solar simulator. The sample holder was cooled to approximately 18.5 °C, measured below the cuvette, to prevent the formation of droplets inside the cuvette due to condensation. The sample cuvette was irradiated for 7 h under Air Mass 1.5 Global (AM1.5G) conditions, for which a spectrum and details of the irradiation power are provided in Figure SI 1 in the Supporting Information. These conditions represent the clear sky solar irradiation at noon in the South of Europe. Therefore, these conditions are also harsher than the expected exposure of the photomolecular heater/UV filter implementations. Fifteen microliters of the irradiated solution was transferred to an LC–MS sample vial to which 745 μL of MeOH was added, making a solution of 20 μM ‘degraded’ SM. Before the IRIS analysis, this sample is used for LC–MS characterization and fractionation.

Using the same cuvette and sample solution, a second irradiation sample was prepared using a Dymax ECE 2000 UV curing lamp, but in this case, the sample was only passively cooled with a strong airflow of 40 m^3^/h and irradiated for 15 min. Power measurements performed with a GaAs reference cell (ReRa Solutions) indicate that in the most relevant short wavelength range (i.e., up to the cut-off wavelength of GaAs at 875 nm), this irradiation is equivalent to approximately 30 h of sun at solar noon. However, it should be noted that this exposure is predominantly in the near-UV and UV parts of the spectrum (see Figure SI 1), whereas the solar spectrum is more evenly distributed. Nevertheless, we feel that this sample provides additional insight into photostability and byproduct generation.

A third sample for which the irradiation step was omitted was prepared as a control sample.

### LC–MS

A Bruker Elute HPLC system was used for sample analysis. The HPLC is equipped with a column oven and autosampler and is coupled to a Bruker AmaZon ion trap mass spectrometer. For high-resolution accurate mass (HRAM) determination, the LC system was instead coupled to a Bruker SolariX Fourier Transform ion cyclotron resonance mass spectrometer. The autosampler was held at 4 °C, while the column oven was held at 40 °C during separation on a Waters Acquity UPLC HSS T3 reversed-phase C18 column with dimensions of 2.1 × 150 mm packed with 1.8 μm particles with a 100 Å pore size on which injections of 2 μL were realized. Elution was performed under a linear gradient from 95% solvent A (0.1% FA in water) and 5% solvent B (0.1% FA in MeOH) at a 0.4 mL/minutes flow rate to the reversed conditions in 15 min. These conditions were held for an additional 5 min before switching back to the initial conditions in 1 min and kept for an additional 5.5 min to allow for equilibration of the column.

For fractioning the analytes of interest, the elution time of the analytes was confirmed using the mass spectrometer. Subsequently, three injections were fractioned by programming the switch valve to divert the flow to a sample vial at the observed elution time of the individual analytes of interest. The acquired samples were stored at 5 °C until use for IRIS analysis, for which the sample was diluted with 250 μL of MeOH with 5% formic acid.

### Infrared Ion Spectroscopy

While the LC–MS analysis provides for a sensitive detection and MS(/MS) characterization of the photoproducts, precise molecular structure determination is limited. We, therefore, obtain IR spectra of the LC–MS-isolated features of interest through the application of IRIS.^40^ Note that the density of mass-selected ions in any (ion trap) mass spectrometer is far too low to obtain a conventional absorption spectrum. Fractionated samples were infused using a Hamilton 250 μL syringe, and the ions of interest were mass-isolated from each LC fraction in the ion trap.^38,39^ The Bruker AmaZon quadrupole ion trap is modified to access the trapped and mass-selected ion cloud with a focused IR laser beam. We employ the FELIX free-electron laser as a radiation source, which is tunable across the entire fingerprint infrared frequency range.^48^ Hardware and software modifications to the AmaZon platform synchronize the MS sequence with the 10-Hz pulse train generated by FELIX. An IRIS spectrum is recorded in situ in the trap by measuring the IR-induced fragmentation yield while scanning the IR laser frequency from 550 to 2250 cm^–1^ in steps of 3 or 5 cm^–1^. The IR-induced fragmentation yield is plotted as a function of the IR frequency to generate an IRIS spectrum, which is generally a good proxy for the IR absorption spectrum. The yield was linearly corrected for frequency-dependent variations in laser pulse energy, and the FELIX laser frequency was calibrated using a grating spectrometer.^49^

### Quantum-Chemical Computation of IR Spectra

A shortlist of potential structures for features of interest in the irradiated sample was generated based on chemical intuition, considering the HRAM data, CID MS/MS fragments, and the IRIS spectrum. Moreover, the molecular structure was assumed to be related to SM. These candidate structures were used as input for a quantum-chemical computation workflow based on the cheminformatics toolbox RDKit in Python 3 and the Gaussian16 quantum chemistry package.^50,51^

The candidate structures are entered in SMILES notation, and all oxygen and nitrogen atoms are considered possible protonation sites or coordination anchors for an Na^+^ ion. Five hundred 3D conformations are randomly generated and energy-minimized employing the MMFF94 force field and then classified by a distance geometry algorithm. Distinct conformations are further geometry-optimized at the semiempirical PM6 level in Gaussian16, followed by vibrational analysis.^51^ The PM6-generated conformers are filtered for duplicates, and structures with broken bonds are discarded. A relative Gibbs free energy cut-off of 40 kJ/mol (at the PM6 level) eliminates structures with unfavorable protonation/Na^+^-anchoring sites. The 20 lowest Gibbs free energy structures are further optimized using the B3LYP density functional and the 6-31++G(d,p) basis set, improving the computed geometries, relative energies, and vibrational spectra compared to PM6. Additionally, a single-point Møller–Plesset second-order (MP2) correction to the Hartree Fock energy is calculated to improve the electronic energy calculation, replacing those established at the B3LYP level.^51^

Harmonic vibrational frequencies are scaled by a factor of 0.975. Calculated stick spectra are convolved with a Gaussian lineshape function of 20 cm^–1^ full width at half maximum to compare the theoretical spectra with those found experimentally. We may obtain multiple low-energy conformations from the conformational search, which could also coexist in the ion population. A weighted-average spectrum is therefore determined, where spectral contributions of different conformers are weighed by their Boltzmann factor at 298.15 K. These Boltzmann-averaged spectra are provided in the SI for all compounds discussed. Although more than one low-energy conformer may thus contribute to the measured IR spectrum of the mass-isolated ion population, we shall propose a single calculated structure for each degradation product, based on the degree of IR spectral matching and relative energy, in order to facilitate an efficient discussion. All spectra were normalized in intensity to facilitate the comparison of experimental and computed spectra.

### In Silico Prediction of Toxicity

For a preliminary assessment of the human and environmental safety of the breakdown products of SM established here, we employ in silico predictions generated through the VEGAHUB platform, which is a low-cost but relatively well-established tool in the environmental safety community.^52^

## Results and Discussion

**Figure 2:**
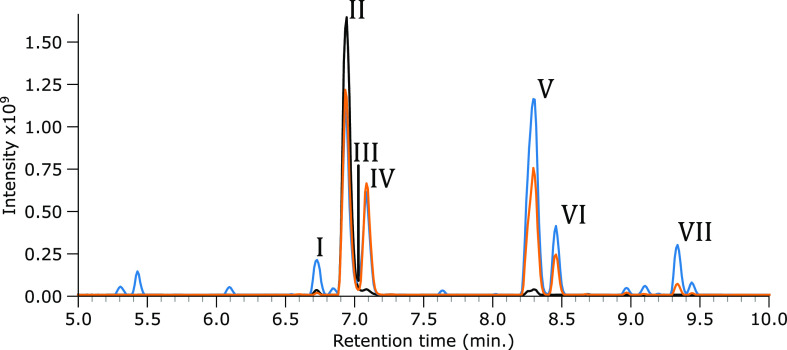
Base-peak chromatograms (BPCs) of three SM samples. Nonirradiated SM (black), SM irradiated with simulated solar radiation for 7 h (orange), and SM irradiated with UV radiation for 15 min (blue). Some chromatographic features are labeled with roman numerals to facilitate their discussion.

As described in the Methods Section, we employed two irradiation sources for the degradation of SM, simulated solar radiation, and harsher UV exposure. LC–MS was used to identify molecular features found after irradiation that were not present in the original sample. Figure 2 depicts three chromatograms with the black trace representing the original sample of SM, the orange trace representing the sample that underwent simulated solar irradiation, and the blue trace representing the sample that underwent harsher UV radiation conditions. The two chromatograms of the irradiated samples show that several degradation products are generated upon irradiation. Additionally, it was noted that some *m*/*z* values of interest appeared more than once in the separation, likely due to multiple structural isomers arising from degradation. The main chromatographic features are labeled with roman numerals to facilitate their discussion in the text. A list of these features, including their HRAM, is provided in Table SI 1. The features of interest were fractionated from the irradiated mixtures for IRIS characterization. Comparison with computationally predicted IR spectra of candidate structures leads to the photoproduced species’ structural identification.

### Isomerization Product of SM at *m*/*z* 363

Figure 2 shows that under simulated solar radiation, the original SM peak (feature II) is reduced in intensity due to degradation, whereas a new peak with high abundance appears as feature IV. The base peak of both chromatographic features is an ion at *m*/*z* 363, which corresponds to the sodiated adduct of SM, as was verified by an HRAM measurement. MS/MS analysis of the *m*/*z* 363 ions from both chromatographic features gives identical fragmentation patterns, as shown in Figure SI 2. Therefore, feature IV corresponds likely to the cis-isomer of SM, as trans/cis isomerization is known to occur upon UV excitation and, in fact, drives the internal conversion upon which the heating action of SM is based.^14,23^

**Figure 3:**
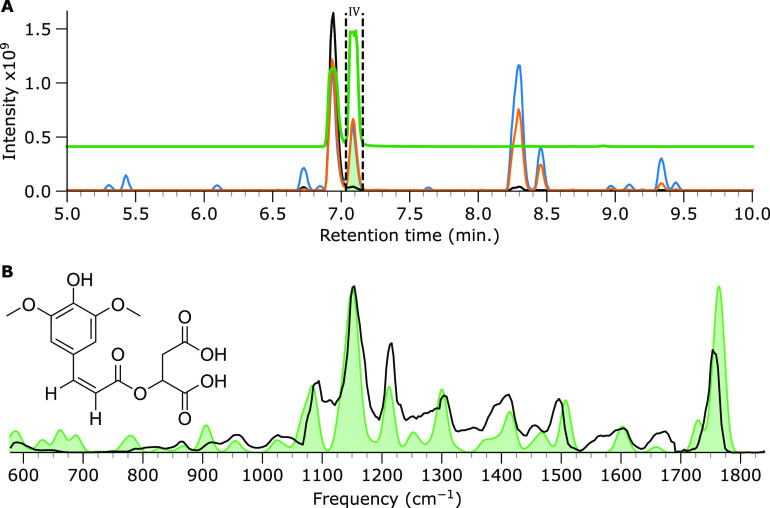
IRIS spectrum of the *m*/*z* 363 ion in the feature IV fraction. (A) BPC of SM (black trace), SM irradiated by simulated solar radiation (orange trace) with vertical dashed lines and green filled curve indicating LC fractioning, SM irradiated by UV (blue trace), and a normalized extracted ion chromatogram (EIC) curve of the *m*/*z* 363 ion (green trace). (B) Measured IRIS spectrum depicted in black with the computed spectrum of the cis isomer of SM (Na^+^ adduct) given as a green-filled curve.

Feature IV was fractioned, as shown in Figure 3A, and the *m*/*z* 363 ion was mass-isolated in the trap. Its IR ion spectrum was recorded, as depicted in Figure 3B, together with the computed IR spectrum of the cis-isomer of SM. A qualitative inspection of the spectra shows that the spectral features and intensities agree favorably between the measured spectrum and computed spectrum. A diagnostic peak occurs at 1660 cm^–1^; an inspection of the computed normal mode vibrations suggests that this band corresponds to the C=C stretch vibration of the cis-isomer. Likewise, the feature at 1766 cm^–1^ comprises the two unresolved C=O stretch bands of the carboxylic acid moieties coordinating with the sodium ion. The band at 1758 cm^–1^ is assigned to the carbonyl stretch of the ester group, which does not coordinate with sodium. Based on these observations and the known trans/cis isomerization, the cis isomerization product is confidently identified as the photodegradation product eluting as feature IV.

### Irradiation Products at *m*/*z* 247 and 471

**Figure 4:**
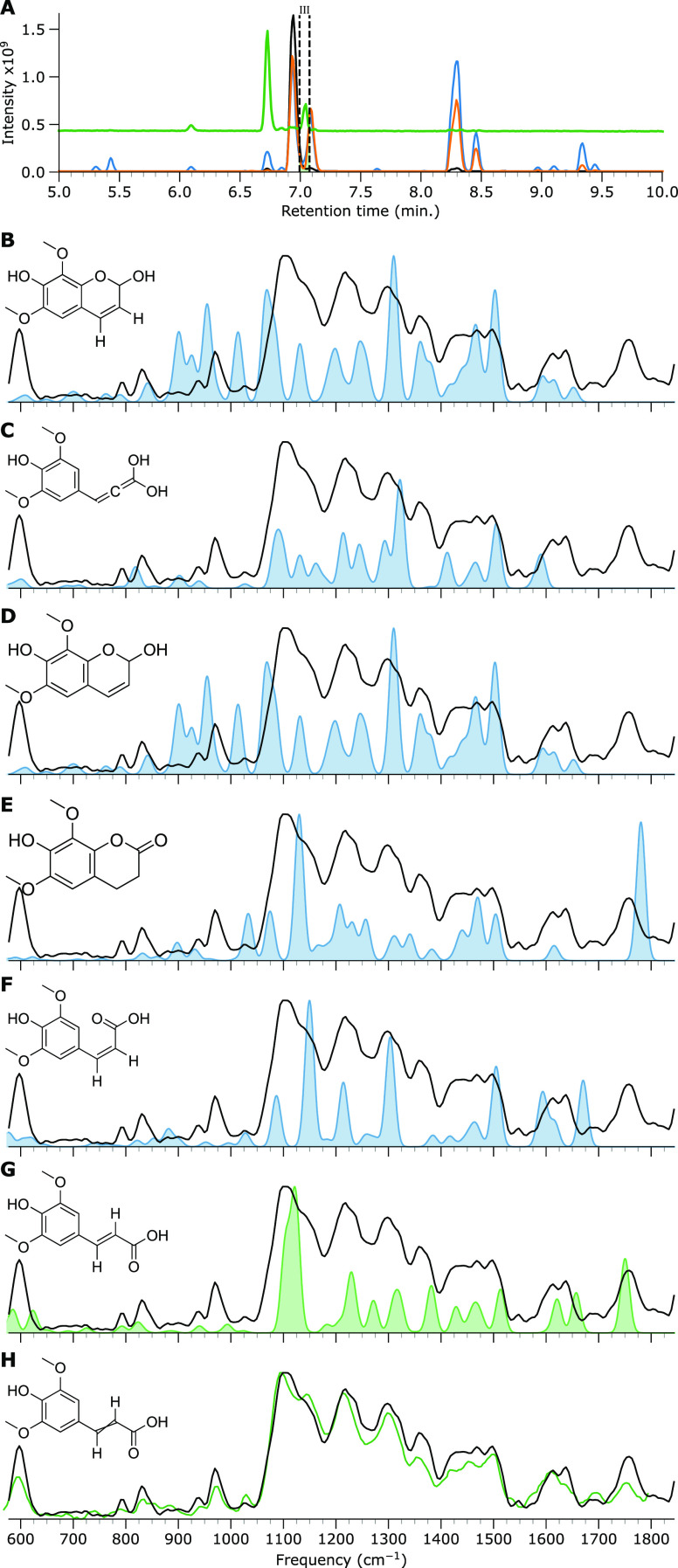
IRIS spectrum and computed IR spectra of the *m*/*z* 247 ion of the chromatographic feature III. (A) BPC of SM (black trace), SM irradiated by simulated solar radiation (orange trace) with vertical dashed lines and green filled curve indicating LC fractioning, SM irradiated by UV (blue trace), and a normalized EIC of the *m*/*z* 247 ion (green trace). Panels B–G depict the measured IRIS spectrum as a black trace and the computed IR spectrum of the sodiated adduct of the candidate structure as a colored trace. H: measured IRIS spectrum of the sodiated adduct of the sinapic acid reference (green trace).

A secondary product at a much lower intensity coeluting with the *m*/*z* 363 chromatographic features is an ion at *m*/*z* 247. Before irradiation, this mass is seen to contribute to LC-feature I as a minor impurity, while after irradiation, it is also observed in feature III; additionally, feature I increases significantly in intensity upon irradiation. LC-feature III was fractionated and infused into the QIT, where the *m*/*z* 247 ion was isolated for IRIS characterization. No IR-induced fragment ions were observed, indicating that their *m*/*z* values are below the low-mass cut-off of the ion trap.^49^ Therefore, the IR ion spectrum was obtained by monitoring the depletion of the *m*/*z* 247 ion signal as a function of the laser frequency (see Figure 4). HRAM analysis determined that the *m*/*z* 247 ion is a sodium adduct of C_11_H_12_O_5_. The experimental IRIS spectrum is compared with computed spectra for the sodium adducts of several candidate compounds. The computed spectrum for the sinapic acid structure in Figure 4G provides the closest match to the measured spectrum. Notably, a feature at 1775 cm^–1^ indicates the presence of a carbonyl group, reproduced in the computed spectra in Figure 4E,G. However, the spectrum in Figure 4E does not reproduce the feature at 600 cm^–1^, where a distinct feature is observed in the IRIS spectrum. Furthermore, the bands between 1100 and 1500 cm^–1^ for sinapic acid in Figure 4G roughly coincide with absorption maxima in the experimentally obtained spectrum, although the latter shows relatively broad and partially unresolved features in this range. Different sinapic acid sodium adduct ion conformers are computationally identified, and their IR spectral fingerprints are averaged after weighing by the Boltzmann factor for their relative Gibbs energy. Figure SI 3 shows that the match in relative intensities and peak shapes improves upon considering all conformers. Predicted spectra for other C_11_H_12_O_5_Na^+^ isomers do not match as well with the experimental spectrum. In order to confirm our assignment, an IRIS spectrum was recorded for the Na^+^-adduct of a reference standard of sinapic acid, which is shown in Figure 4H. The spectrum of the external reference standard matches the sample spectrum exactly, confirming the identity of the fractioned *m*/*z* 247 feature as sinapic acid.

Sinapic acid can be formed by ester hydrolysis of SM, and although its production from SM should occur spontaneously over time in the solution, the production rate appears to be vastly accelerated by irradiation. We note that the external standard is a mixture of cis and trans sinapic acid, which matches the fractioned chromatographic peak showing a chromatographic separation of 0.4 min. This suggests that the fractionation process isolates a pure fraction. Nevertheless, we can only explain the shoulder feature of the unresolved feature in the experimental IR spectrum at 1125 cm^–1^ by the feature of the cis isomerized sinapic acid at 1150 cm^–1^, attributed to an OH bending mode of the carboxylic acid in Figure 4F. This implies that some isomerization occurs between fractioning the chromatographic peak and the IRIS characterization. Alternatively, this deviation may be due to small errors in the density functional theory (DFT) computation of the spectra.

A secondary ion at *m*/*z* 471 is observed with an elution profile identical to sinapic acid. However, this second ion is only generated after UV irradiation. Only a poor-quality IRIS spectrum for the *m*/*z* 471 ion could be obtained, but its structure can be assessed based on the HRAM mass and the MS/MS fragmentation spectra. The fragmentation mass spectrum of *m*/*z* 471, as depicted in Figure SI 4, contains a fragment at *m*/*z* 247. Furthermore, the HRAM mass predicts a chemical formula equivalent to a sodium-bound dimer of sinapic acid. Based on these observations, we can confidently assign the *m*/*z* 471 ion as the sodium-bound dimer of the IRIS-identified sinapic acid.

### Fragment Ion at *m*/*z* 207

**Figure 5:**
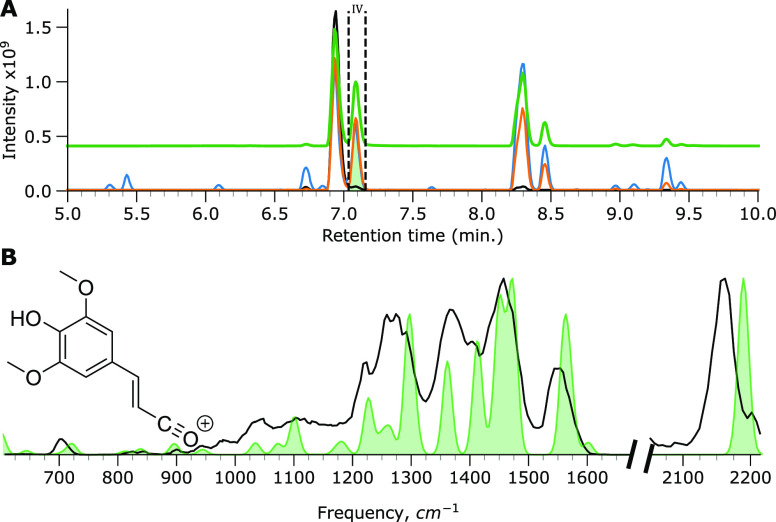
IRIS spectrum and computed IR spectra of the *m*/*z* 207 ion in LC-feature IV. (A) BPC of SM (black trace), SM irradiated by simulated solar radiation (orange trace) with vertical dashed lines and green filled curve indicating LC fractioning, SM irradiated by UV (blue trace), and a normalized EIC curve of the *m*/*z* 207 ion (green trace). (B) Measured IRIS spectrum depicted in black with the computed spectrum of the ester-cleaved acylium fragment ion of trans-SM given as a green-filled curve.

It was observed that several chromatographic peaks contained a feature at *m*/*z* 207, whose intensity correlated with the base peak. Upon inspection of the HRAM mass spectra, the feature did not appear to have any adduct mass peaks in the spectrum, indicating that it could originate from (in-source) fragmentation. Based on these observations, an acylium fragment ion structure shown in Figure 5B is suggested. A fraction was obtained from chromatographic feature IV to evaluate the origin of the *m*/*z* 207 ion, as depicted in Figure 5A. The IR ion spectrum was recorded and is shown in Figure 5B, along with the computed IR spectrum of the acylium ion structure, providing a reasonably convincing match. A particularly diagnostic feature is the intense acylium C≡O^+^ stretch vibration predicted at 2190 cm^–1^, previously observed for other gas-phase acylium ion-containing species, such as cationic benzoyl derivatives.^53,54^ The deviation in the peak position between measured and computed spectra is similar to what has been observed for these benzoyl derivatives; moreover, a triple-bond species is likely as this spectral range is otherwise usually devoid of features. Furthermore, the overall shape of the remainder of the spectrum matches well with the predicted spectrum for the acylium compound. We further note that the in-source formation of acylium ions is an established process.^55,56^

Efforts were made to measure multiple fractions to identify cis and trans isomers of the *m*/*z* 207 ion, but the differences in the computed spectra of the two isomers are too small to differentiate them. Based on this assignment, we assume that the product is an in-source fragmentation product and would not require extensive human and environmental toxicity evaluation as it is likely not present in the solution.

### Esterification Product Ions at *m*/*z* 377 and 391

**Figure 6:**
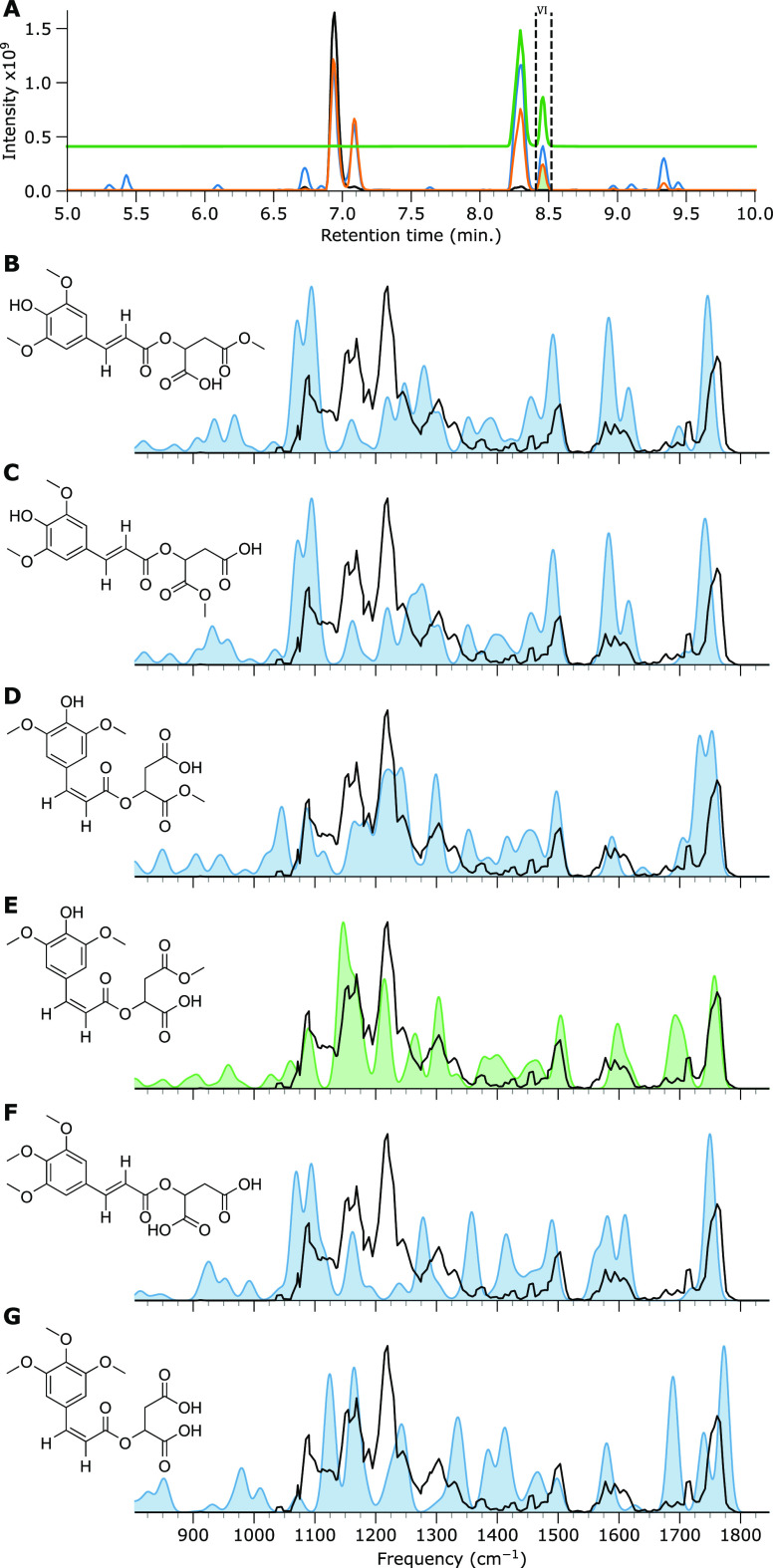
IRIS spectrum and computed IR spectra of the 377 *m*/*z* ion of feature VI. (A) BPC of SM (black trace), SM irradiated by simulated solar radiation (orange trace) with vertical dashed lines and green filled curve indicating LC fractioning, SM irradiated by UV (blue trace), and a normalized EIC curve of the *m*/*z* 377 ion (green trace). All remaining panels depict the measured IRIS spectrum as a black trace, and the computed IR spectrum of the sodiated adduct of the candidate molecule is shown as a colored curve (panels B–G).

**Figure 7:**
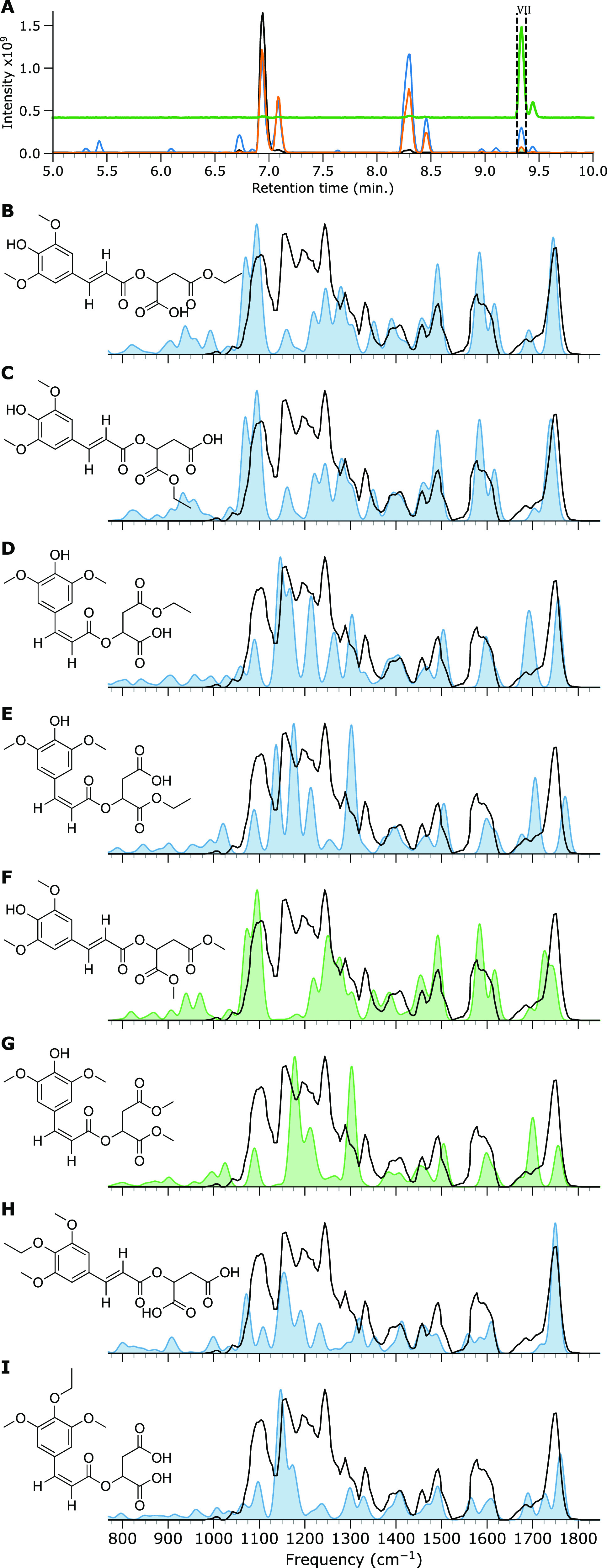
IRIS spectrum and computed IR spectra of the *m*/*z* 391 ion of feature VII. (A) BPC of SM (black trace), SM irradiated by simulated solar radiation (orange trace) with vertical dashed lines and green filled curve indicating LC fractioning, SM irradiated by UV (blue trace), and a normalized EIC curve of the *m*/*z* 391 ion (green trace). All remaining panels depict the measured IRIS spectrum as a black trace, and the computed IR spectrum of the sodiated adduct of the candidate molecule is shown as a colored curve (panels B–I).

Two byproducts found after irradiation have a mass that is higher than SM, the ions at *m*/*z* 377 and 391. When the chromatographic profiles of both features are evaluated, it is noted that both ions appear in two chromatographic features, as can be observed in Table SI 1. Their HRAM suggests molecular formulas that fit with sodiated adducts of alkylated derivatives of SM. Considering the structure of SM, one possibility arises from the esterification of the carboxylic acids and the other from the less likely alkylation of the hydroxyl group present on the syringol moiety. For the *m*/*z* 391 ion, two types of esterification products are possible; the first is a dimethyl-SM ester, and the second is an ethyl ester resulting from the esterification of one of the two carboxylic acid groups. Esterification of carboxylic acids by alcohols is an established process, and since methanol is used as a solvent rather than ethanol, methylated products are deemed more likely than ethylated ones. We also consider that all products could be cis or trans isomerized, leading to eight possible isomers for the *m*/*z* 391 ion. For the *m*/*z* 377 ion, only a single carboxylic acid has undergone esterification leading to six candidate structures (methanol addition to one of three OH-moieties and either cis or trans). We collected a fraction of features V and VI from the irradiated SM sample for IRIS analysis. In both cases, the IR ion spectrum was recorded for the sodium adduct, which is shown in Figures 6 and 7 for the *m*/*z* 377 and 391 ions, respectively.

We first discuss the *m*/*z* 377 ion, for which a qualitative comparison with computed spectra in Figure 6D,E suggests that the cis isomers of the esterification products provide a better match than the trans isomers (Figure 6B,C). Specifically, the feature at 1600 cm^–1^ attributed to the C=C stretch vibration, which is distinct in cis and trans isomers, agrees favorably with the computed spectra of the cis isomers. For the trans isomers, the feature is split into a doublet, whereas in the cis isomers, the feature is predicted as a lower-intensity singlet. The differentiating features for the computed spectra of Figure 6D,E are in the region between 1000 and 1100 cm^–1^ and between 1700 and 1800 cm^–1^. In the first region, Figure 6D shows a predicted doublet band, while the predicted spectrum of Figure 6E presents a singlet. The remaining band in this region at 1075 cm^–1^, which is predicted for both structures, is assigned to an in-plane OH bending vibration of the syringol moiety. Additionally, we note that the features between 1650 and 1775 cm^–1^ appear to agree more favorably with the structure of Figure 6D than with that of Figure 6E. However, the feature predicted at 1725 cm^–1^ in Figure 6D, attributed to a Na^+^-coordinated carbonyl stretch vibration of the two formerly carboxylic acid carbonyls of SM, deviates significantly from the experiment, whereas such carboxylic stretches are usually well predicted by B3LYP. The same spectral region in Figure 6E matches both position and relative intensity favorably. However, the intensity of two ester carboxyl vibrations at 1700 cm^–1^ appears underrepresented in the experimental spectrum compared to the predicted spectrum in Figure 6E. When we examine the Boltzmann-weighted average spectrum of all conformers in Figure SI 5, we note an improved match in intensity. Both the syringol moiety alkylation products (Figure 6F,G) can be discarded based on the measured feature at 1225 cm^–1^, which is not present in the computed spectrum.

We, therefore, assign the structure shown in Figure 6E to the fractioned chromatographic peak in Figure 6A. However, the EIC at *m*/*z* 377 consists of multiple, closely spaced features (see Figures 6A and SI 6), so that we suspect that all four esterification products are formed upon irradiation of SM. Similar esterification reactions may occur when SM is used as a photomolecular heater on crops, but this specific product results from the MeOH used as a solvent here. We further note that the IRIS analysis and assignment of the methyl carboxylic acid esterification product were done for the chromatographic feature of highest abundance, suggesting that this is the preferential product.

With the tentative assignment of the *m*/*z* 377 ion, we now focus on the *m*/*z* 391 ion. Also, here, esterification products are the most likely candidates. Furthermore, we note that the *m*/*z* 377 ion could act as an intermediary product for the dimethyl esterification of SM. The structures and DFT-predicted IR spectra of the eight suggested alkylation products of *m*/*z* 391 are depicted in Figure 7. Upon examining the eight spectra, none of them stands out as an excellent match by itself. Therefore, despite our efforts to cut pure fractions from the chromatographic analysis, it is not unlikely that the ion population contains more than one structural isomer. Alternatively, isomerization after fractioning may occur, as discussed above, for the *m*/*z* 247 ion. Nonetheless, we can arrive at a tentative assignment by eliminating compounds based on spectral mismatches.

The predicted spectra of the syringol-alkylated isomers in Figure 7H,I lack a feature at 1250 cm^–1^, which is dominant in the experimental spectrum. Therefore, these two products are considered unlikely, leaving six possible esterification products. Of these six, the computed spectra of the trans-isomers in Figure 7B,C,F best reproduce the 1250 cm^–1^ feature. In contrast, the cis-isomers’ spectra in Figure 7D,E,G appear to lack a clear feature at this position. Moreover, all cis-isomers are predicted to possess an intense feature at 1700 cm^–1^, but the measured spectrum shows only a weak absorption at this position, which is better in line with the predicted spectra for the trans-isomers. Therefore, we suggest that the measured spectrum is predominantly due to a trans-isomerized esterified SM.

Considering the carboxylic C=O stretch vibrations around 1750 cm^–1^, the predicted spectra of Figure 7B,C match well with the measured spectrum, whereas the spectrum of Figure 7F shows a minimal deviation. None of the three isomers appear to match the peak pattern in the 1150–1250 cm^–1^ range particularly well, although taking the Boltzmann-weighted average spectrum of all conformers (see Figure SI 7) suggests that the ethyl ester in Figure 7C provides the best match. On the other hand, considering that the solution contained methanol but not ethanol, a dimethyl ester is more likely than an ethyl ester. Looking at the computed spectra for the cis (Figure 7G) and trans (Figure 7F) isomers of the dimethyl ester, a mixture of both species could also plausibly explain the measured IRIS spectrum if we accept a slight mismatch of the band computed near 1160 cm^–1^. We shall adopt this latter assignment, although it is tentative at best.

## Concluding Remarks

An overview of the UV-induced transformation products of SM identified in this study is provided in Table 1. We conclude that the nature of the major byproducts can be classified as ester cleavage and esterification products. The esterification products appear to originate from the methanol used to dissolve SM for the experiments. In the formulation used for the foliar spray containing SM, these esterification products are unlikely to form if no methanol is present. However, one may expect analogous products when other alcohols are included in the formulation. The ester cleavage products are expected to be independent of the formulation in which SM is applied. Compared to other bonds in the SM molecule, the more polarized single bonds of the ester linkage are natural targets for reactions. Hence, in retrospect, ester cleavage and esterification products are not unreasonable, given the chemical structure of SM. Nonetheless, these byproducts would have remained tentative without the IRIS measurements and without the appropriate reference standards.

**Table 1:** Overview of Identified Compounds^a^

*m*/*z*	feature ID	Ret. (min)	IUPAC name & trivial name
**363**	**IV**	**7.1**	**(Z)-2-((3-(4-hydroxy-3,5-dimethoxyphenyl)acryloyl)oxy)succinic acid**
			***cis-sinapoyl malate***
**377**	**VI**	8.5	**(Z)-2-((3-(4-hydroxy-3,5-dimethoxyphenyl)acryloyl)oxy)-4-methoxy-4-oxobutanoic acid**
	VI	8.5*	(E)-2-((3-(4-hydroxy-3,5-dimethoxyphenyl)acryloyl)oxy)-4-methoxy-4-oxobutanoic acid
	V	8.3*	(Z)-3-((3-(4-hydroxy-3,5-dimethoxyphenyl)acryloyl)oxy)-4-methoxy-4-oxobutanoic acid
	V	8.3*	(E)-3-((3-(4-hydroxy-3,5-dimethoxyphenyl)acryloyl)oxy)-4-methoxy-4-oxobutanoic acid
**391**	**VII**	**9.3**	**dimethyl (E)-2-((3-(4-hydroxy-3,5-dimethoxyphenyl)acryloyl)oxy)succinate**
			***trans-dimethyl sinapoyl malate***
	VII	9.4*	dimethyl (Z)-2-((3-(4-hydroxy-3,5-dimethoxyphenyl)acryloyl)oxy)succinate
			*cis-dimethyl sinapoyl malate*
**247**	**III**	**7.0**	**(E)-3-(4-hydroxy-3,5-dimethoxyphenyl)acrylic acid**
			***trans-sinapic acid***
	I	6.7*	(Z)-3-(4-hydroxy-3,5-dimethoxyphenyl)acrylic acid
			*cis-sinapic acid*
**207**	**multiple**	multiple	**4-(3-(l3-oxidaneylidyne)prop-1-en-1-yl)-2,6-dimethoxyphenol**
			**sinapaldehyde (neutral)**

a Compounds in bold denote those identified with IRIS, whereas those marked with ‘*’ indicate those elucidated as isomers of identified compounds.

With the structural characterization of the UV-breakdown products of the photomolecular heater established, we can perform a preliminary assessment of their potential toxicity. Disregarding the cis/trans isomerization, we used the VEGAHUB in silico toxicity predictor to obtain an indication of their human and environmental safety. We note that sinapic aldehyde, measured here as the oxonium ion, and sinapic acid are known flavoring agents and thus pose little concern regarding human and environmental toxicity. The results of the VEGAHUB analysis of all byproducts established here are presented in Table SI 2 and suggest that no significant concern is expected regarding human and environmental safety compared to other agrochemical compounds. Nevertheless, for applying SM as a photomolecular heater, more detailed regulatory studies regarding human and environmental safety and aquatic toxicity will be necessary.

As a more general result, we have demonstrated that the identification of UV-photodegradation products can be quickly narrowed down in a reference-free manner using the combination of LC–MS/MS, IRIS, and DFT. Spectral matching to external reference standards can then confirm the reference-free tentative assignments. The identifications were made here using minimal sample amounts of 6 μL for all identified features of interest, which derives from the fact that IRIS provides IR spectral fingerprints with the sensitivity of (LC−)MS. The ability to select a product by fractionation allows for a more straightforward interpretation of a single species when multiple isomers may be present in the sample matrix. This selectivity and sensitivity may aid in identifying agrochemical transformation products in cases where NMR structure elucidation is impossible because of low concentrations and/or complex sample matrices. Eventually, analyses of agrochemical degradation products in field trials will enable the development of target-oriented agrochemicals that, on the one hand, are optimized for their specific purpose but, on the other hand, have a minimized degradation profile.

## Supporting Information Available

UV–vis spectra of irradiation sources used, high-resolution accurate mass data for LC–MS peaks of interest. CID MS/MS spectra for sinapoyl malate and for the *m*/*z* 471 ion in LC feature III, additional experimental versus theoretical IR spectrum comparisons, including Boltzmann-weighted average theoretical IR spectra, additional LC–MS analysis of irradiated sample performed at Warwick University, and table with results of preliminary in silico toxicity analysis (PDF)
