# Supplementary material for: Characterization of Solar Radiation-Induced Degradation Products of the Plant Sunscreen Sinapoyl Malate
Source: ACS Agric Sci Technol. 2023 Jan 19;3(2):171–80. doi: 10.1021/acsagscitech.2c00279 (PMC9945346; doi:10.1021/acsagscitech.2c00279)
Supplement: Supplementary file 1 — as2c00279_si_001.pdf [file as2c00279_si_001.pdf]

Characterization of Solar Radiation-induced Degradation Products of the Plant Sunscreen Sinapoyl Malate

Matthias J.A. Vink[1], John J. Schermer[2], Jonathan Martens[1], Wybren Jan Buma[1,3], Giel Berden[1*], Jos Oomens[1*]

[1]Radboud University, Institute for Molecules and Materials, FELIX Laboratory, Toernooiveld 7, 6525 ED Nijmegen, The Netherlands

[2]Radboud University, Institute for Molecules and Materials, Heyendaalseweg 135, 6525 AJ Nijmegen, The Netherlands

[3]University of Amsterdam, van 't Hoff Institute for Molecular Sciences, Science Park 904, 1098 XH Amsterdam, The Netherlands

*Corresponding authors: jos.oomens@ru.nl, giel.berden@ru.nl

Supporting Information

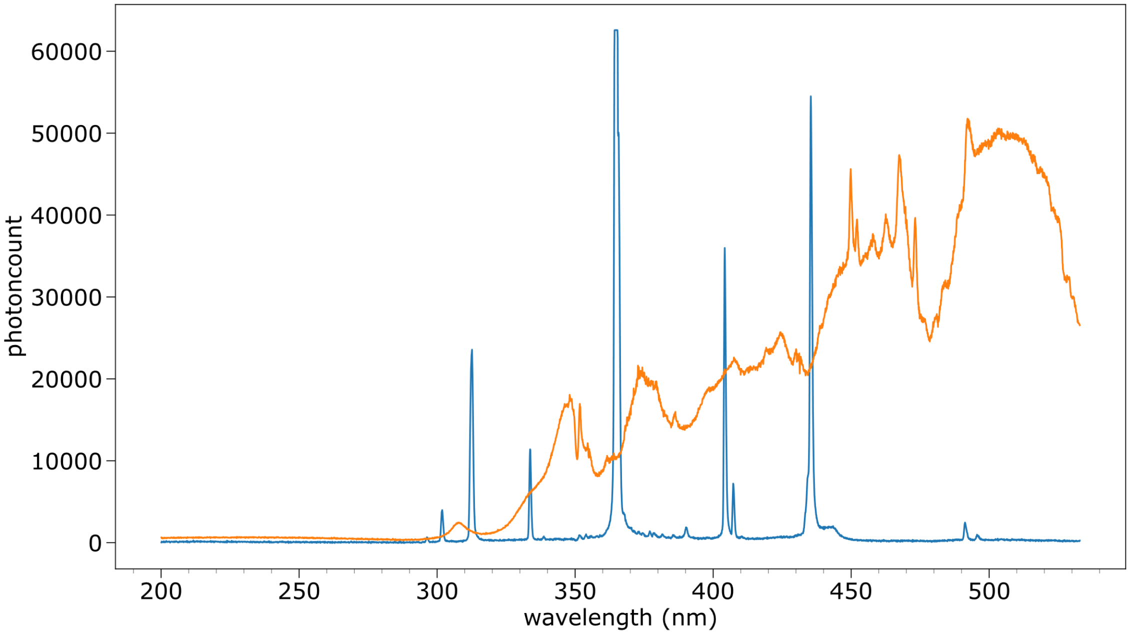

Figure SI 1| Sources for simulated solar irradiation. *Blue curve: Measured spectrum of UV lamp (Dymax ECE 2000 UV curing lamp). Orange curve: Measured spectrum of ABET Technologies Sun 2000 solar simulator lamp (AM1.5 Global). The ABET Technologies Sun 2000 solar simulator is used for irradiation experiments and provides a 0.96 solar equivalent power spectrum. This lamp has a broader spectrum that correlates well with the actual solar spectrum, as compared to the line spectrum generated by the Dymax ECE 2000 UV curing lamp. It should be noted that the spectra have been attenuated such that they can be represented next to each other in the same figure.*

HRAM characterization of ions of interest

Table SI 1 *High-resolution accurate mass feature list of ions of interest after chromatographic separation*

Feature ID	Ret. (min.)		Measured m/z	Theoretical *m/z*
IV	7.1	$C_{15}H_{16}O_9$ $[Na]^+$	363.06954	363.069205
V	8.3	$C_{16}H_{18}O_9$ $[Na]^+$	377.08535	377.084855
V	8.3*			
VI	8.5*		377.08463	377.084855
VI	8.5*			
VII	9.3	$C_{17}H_{20}O_9$ $[Na]^+$	391.1005	391.100505
VII	9.4*		391.0009	
III	7.0	$C_{11}H_{12}O_5$ $[Na]^+$	247.15424	247.058245
I	6.7*		247.05774	
multiple	multiple	$C_{11}H_{11}O_4^+$	207.06538	207.065735

Fragment analysis of SM isomers

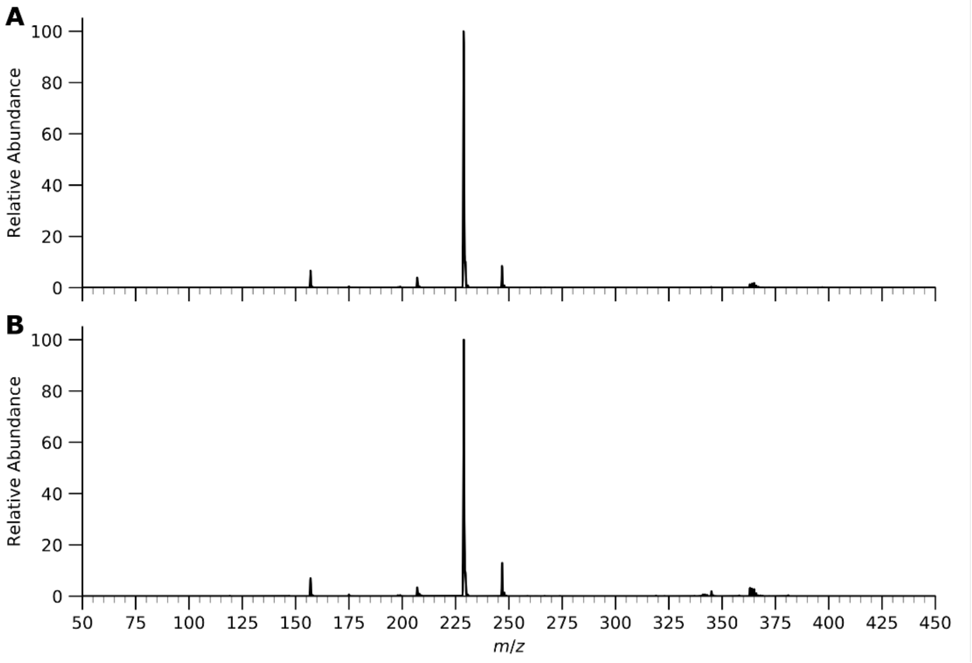

Figure SI 2 | Fragmentation mass spectra of the sodium adducts of SM. ***A****: fragmentation spectrum of trans-SM before simulated solar irradiation.* ***B****: fragmentation spectrum of, from IRMPD analysis of the cis-SM, which is only present after irradiation.*

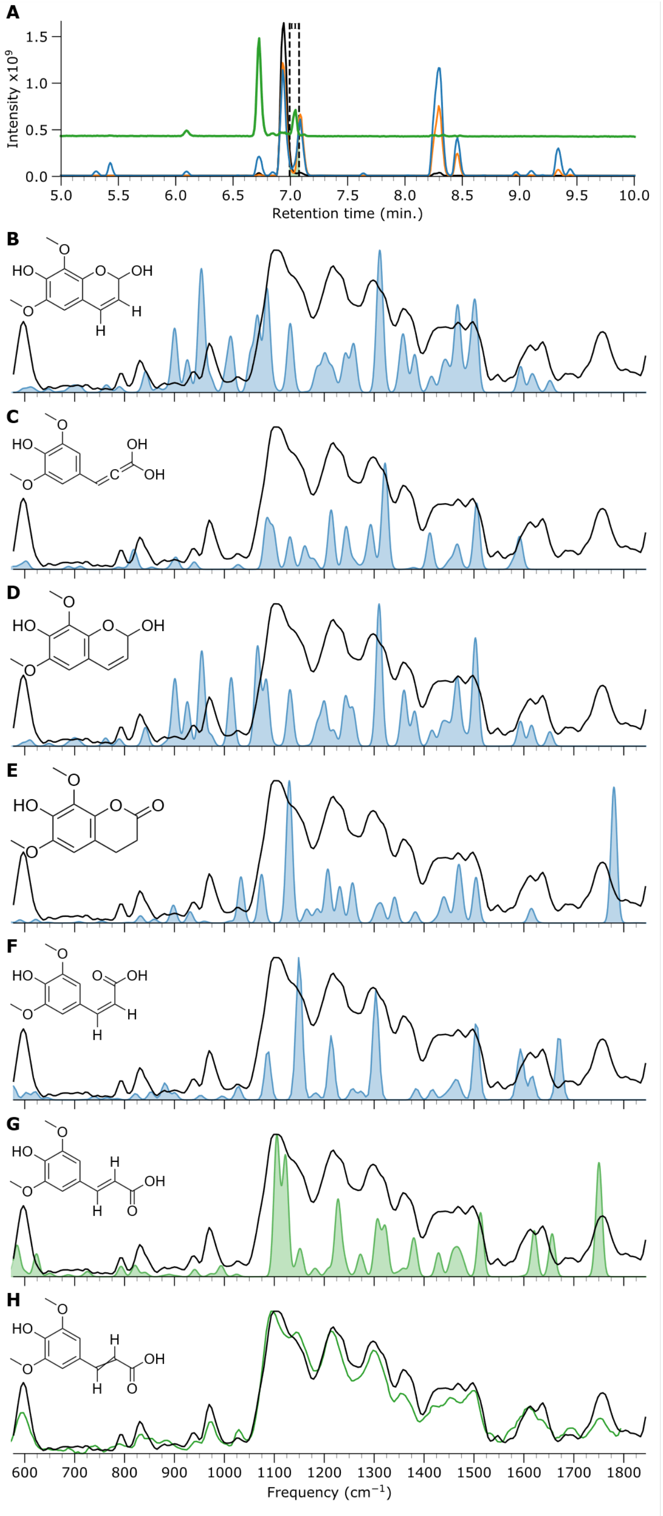

Figure SI 3 | Computed IR spectra of the m/z 247 ion of the chromatographic feature III. ***A****: BPC chromatogram of SM (black trace), SM irradiated by simulated solar radiation (blue trace) with vertical dashed lines and blue filled curve indicating LC fractioning, SM irradiated by UV (red trace), and a normalized EIC curve of the m/z 247 ion (orange trace).* ***Panels B – G*** *depict the measured IRIS spectrum as a black trace and the computed IR spectrum (Boltzmann-weighted average over conformers) of the sodiated adduct of the shown candidate molecule as a colored filled curve.* ***H****: measured IRIS spectrum of sinapic acid reference compound (green).*

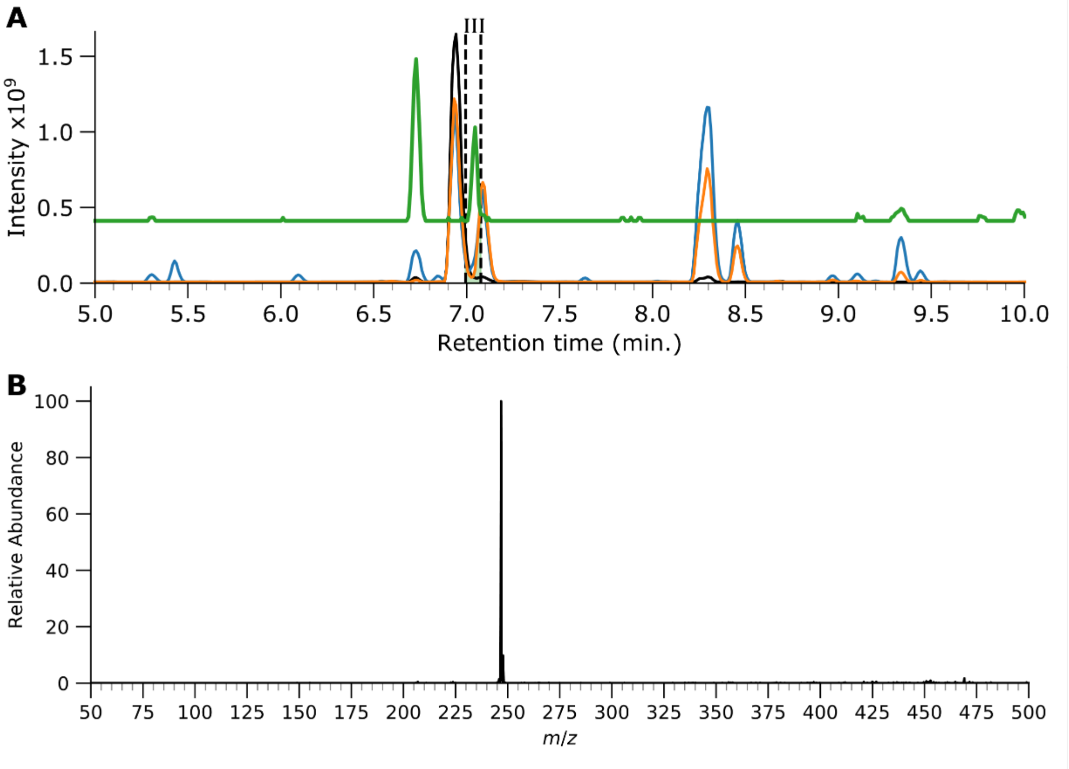

Figure SI 4 | Fragmentation analysis of the *m/z* 471 ion chromatographic feature III. *A*: *BPC chromatogram of SM (black trace), SM irradiated by simulated solar radiation (orange trace with vertical dashed lines and blue filled curve indicating LC fractioning), SM irradiated by UV (blue trace), and a normalized extracted ion chromatogram (EIC) curve of the m/z 363 ion (green trace).* ***B***: *MS/MS spectrum of the m/z 471 ion, producing a single fragment ion at m/z 247.*

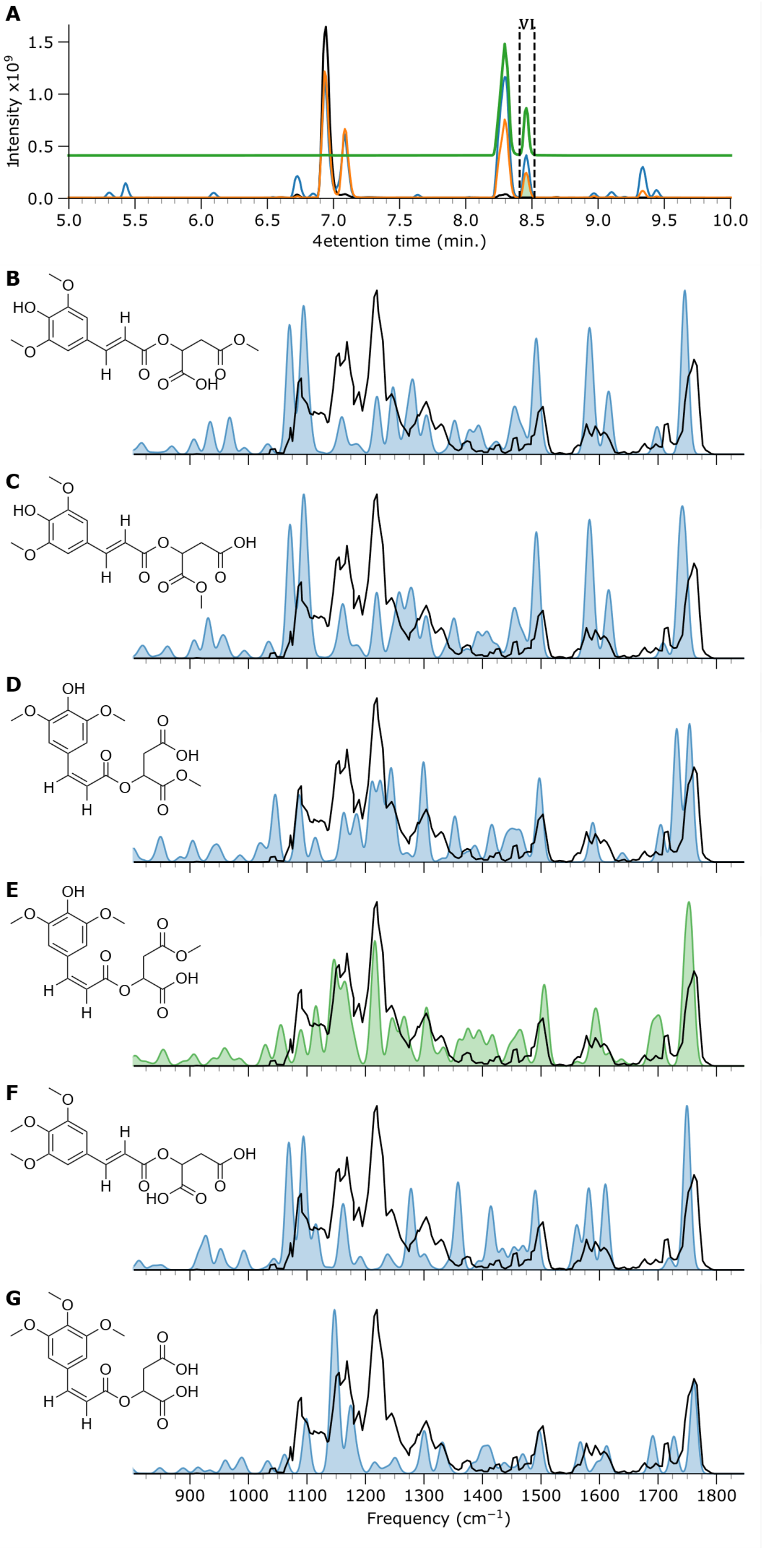

Figure SI 5 | Computed IR spectra of the *m/z* 377 ion chromatographic feature VI. ***A***: *BPC chromatogram of SM (black trace), SM irradiated by simulated solar radiation (blue trace) with vertical dashed lines and blue filled curve indicating LC fractioning, SM irradiated by UV (red trace), and a normalized EIC curve of the m/z 377 ion (orange trace). The* ***remaining panels*** *depict the measured IRIS spectrum as a black trace and the computed IR spectra (Boltzmann weighted average of conformers) of the sodiated adduct of the indicated candidate molecule as a colored filled curve.*

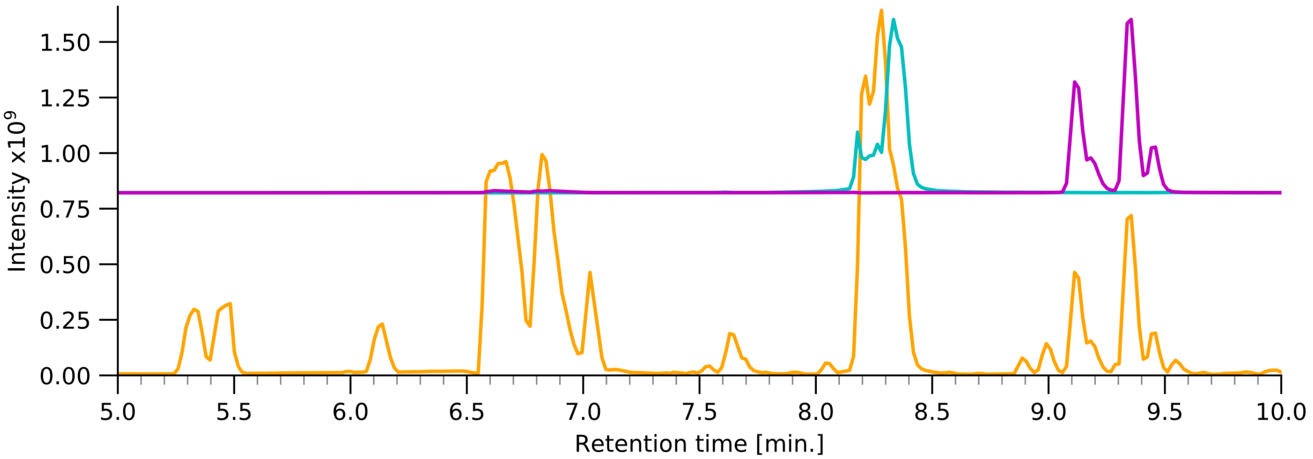

Figure SI 6 | Chromatographic analysis of UV irradiated SM. *Yellow trace is the BPC of an 8-h irradiated SM sample (irradiation performed at Warwick). Cyan trace is EIC of m/z 377, purple trace is EIC of m/z 391.*

Alternative chromatographic analysis

Figure SI 6 shows a chromatographic analysis recorded with the Bruker FTICR-MS of a sample that was irradiated at Warwick University in a 1 cm pathlength quartz cuvette. No air remained in the cuvette for irradiation with a solar simulator (Oriel Instruments, 91191-1000) using neutral density filter to achieve an irradiation power equivalent to one sun (~1000 W/m^2). For the *m/z* 391 ion eluting between 9 and 9.5 minutes, multiple, partially unresolved peaks are observed. Further, a distortion of the peak of the *m/z* 377 ion can be observed, indicating that potentially multiple species co-elute.

Table SI 2 *Summary of in silico toxicity predictions provided by VEGAHUB. For each parameter (except mutagenicity), the result of the in silico analysis is given as well as indication on the reliability of the prediction (low, moderate, good).*

	(E)-2-((3-(4-hydroxy-3,5-dimethoxyphenyl)acryloyl)oxy)succinic acid	(Z)-2-((3-(4-hydroxy-3,5-dimethoxyphenyl)acryloyl)oxy)-4-methoxy-4-	Dimethyl (E)-2-((3-(4-hydroxy-3,5-dimethoxyphenyl)acryl	(E)-3-(4-hydroxy-3,5-dimethoxyphenyl)acrylic acid[1]	(E)-3-(4-hydroxy-3,5-dimethoxyphenyl)acrylaldehyde[2]	(Z)-(3-(4-hydroxy-3,5-dimethoxyphenyl)allylidyne)oxonium
Mutagenicity (Ames test) CONSENSUS model 1.0.3	negative	negative	negative	negative	negative	negative
Estrogen Receptor Relative Binding Affinity model (IRFMN) 1.0.1	active (low)	active (low)	active (low)	active (low)	inactive (low)	inactive (low)
Estrogen Receptor-mediated effect (IRFMN/CERAPP) 1.0.0	non-active (good)	non-active (good)	non-active (good)	possible non-active (moderate)	possible non-active (good)	n.a
Androgen Receptor-mediated effect (IRFMN/COMPARA) 1.0.0	non-active (moderate)	non-active (moderate)	non-active (moderate)	active (low)	active (low)	active (low)
Thyroid Receptor Alpha effect (NRMEA) 1.0.0	inactive (good)	inactive (good)	inactive (good)	inactive (good)	inactive (good)	inactive (low)
Thyroid Receptor Beta effect (NRMEA) 1.0.0	inactive (good)	inactive (good)	inactive (good)	inactive (good)	inactive (good)	inactive (low)
Acute toxicity (rat oral LD50)*	3380 mg/kg	3121 mg/kg	2227 mg/kg	4173 mg/kg	1982 mg/kg	n.a
BCF model (KNN/Read-Across) 1.1.0**	0.84 (moderate)	1.31 (low)	1.28 (low)	0.78 (moderate)	1.19 (low)	0.73 (low)
Ready Biodegradability model (IRFMN) 1.0.9	readily biodegradable (moderate)	readily biodegradable (moderate)	readily biodegradable (moderate)	possible readily biodegradable (moderate)	possible readily biodegradable (moderate)	possible readily biodegradable (low)
Persistence (sediment) quantitative model (IRFMN) 1.0.0	227 days (low)	227 days (low)	227 days (low)	156 days (low)	156 days (low)	156 days (low)
Persistence (soil) model (IRFMN) 1.0.0	not persistent (moderate)	not persistent (moderate)	not persistent (moderate)	not persistent (moderate)	not persistent (moderate)	not persistent (low)
Persistence (water) model (IRFMN) 1.0.0	not persistent (low)	not persistent (moderate)	not persistent (low)	not persistent (moderate)	not persistent (moderate)	not persistent (low)

*[1,2] registered as a food additive (flavoring agent), n.a : not applicable, *prediction provided by TEST (consensus method), **expressed as a log value*

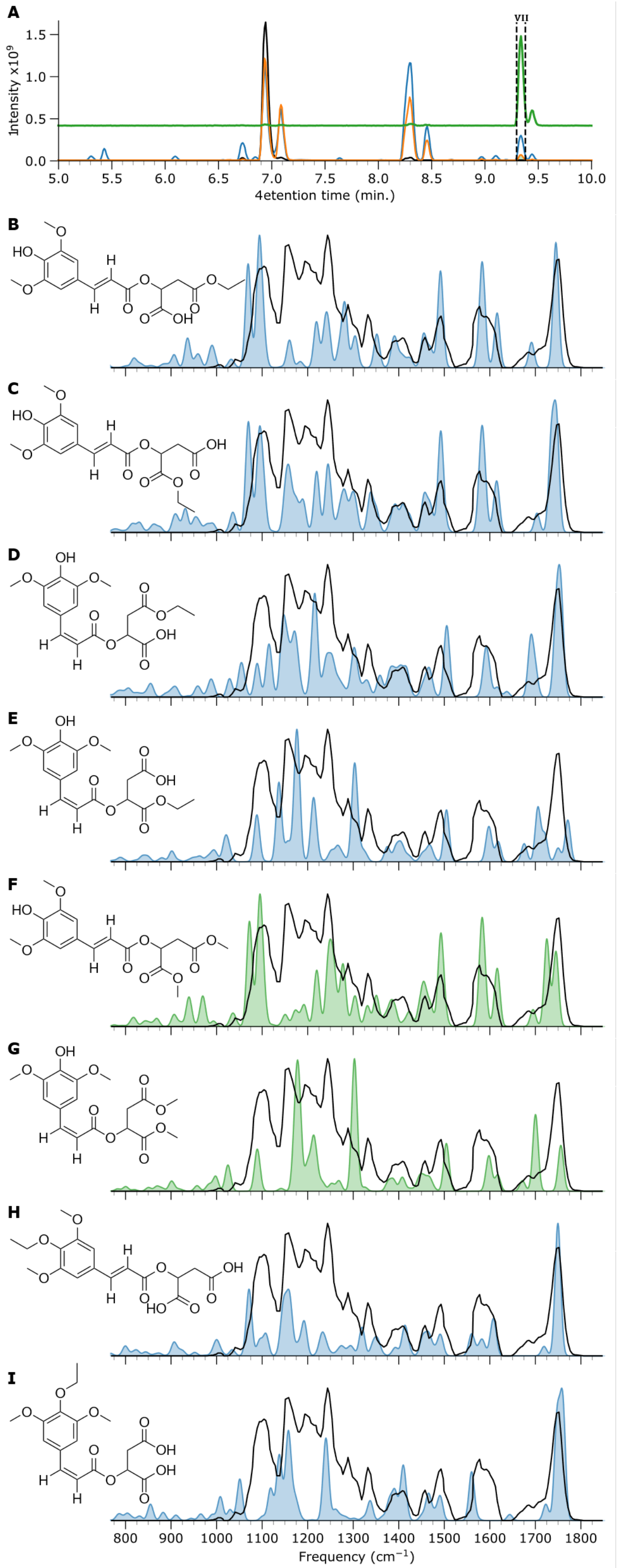

Figure SI 7| IRIS spectrum and computed IR spectra of the m/z 391 ion chromatographic feature VII. ***A****: BPC chromatogram of SM (black trace), SM irradiated by simulated solar radiation (blue trace) with vertical dashed lines and blue filled curve indicating LC fractioning), SM irradiated by UV (red trace), and a normalized EIC curve of the m/z 391 ion (orange trace). All remaining panels depict the measured IRIS spectrum as a black trace and a Boltzmann averaged computed IR spectrum of a sodiated adduct of a candidate molecule as a colored filled curve.*

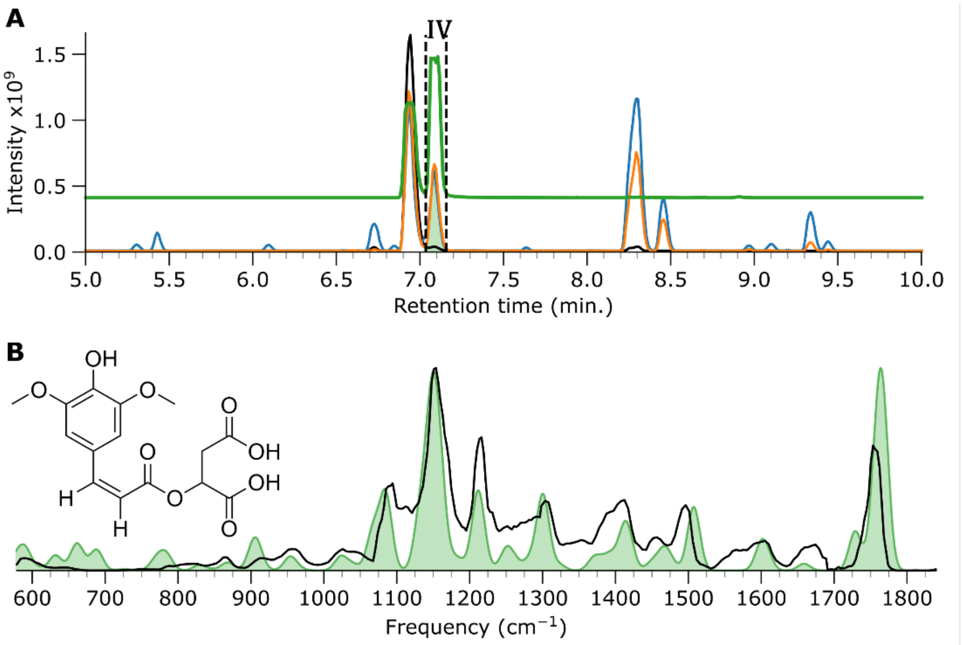

Figure SI 8 | *Boltzmann spectrum of the m/z 363 ion fractioned from* chromatographic feature IV*.* *A**:*** *BPC chromatogram of SM (black trace), SM irradiated by simulated solar radiation (orange trace) with vertical dashed lines and blue filled curve indicating LC fractioning), SM irradiated by UV (blue trace), and a normalized extracted ion chromatogram (EIC) curve of the m/z 363 ion (green trace).* ***B****: Measured IRIS spectrum depicted in black with the Boltzmann averaged computed spectrum of the cis isomer of SM (Na^+ adduct) given as a green filled curve.*

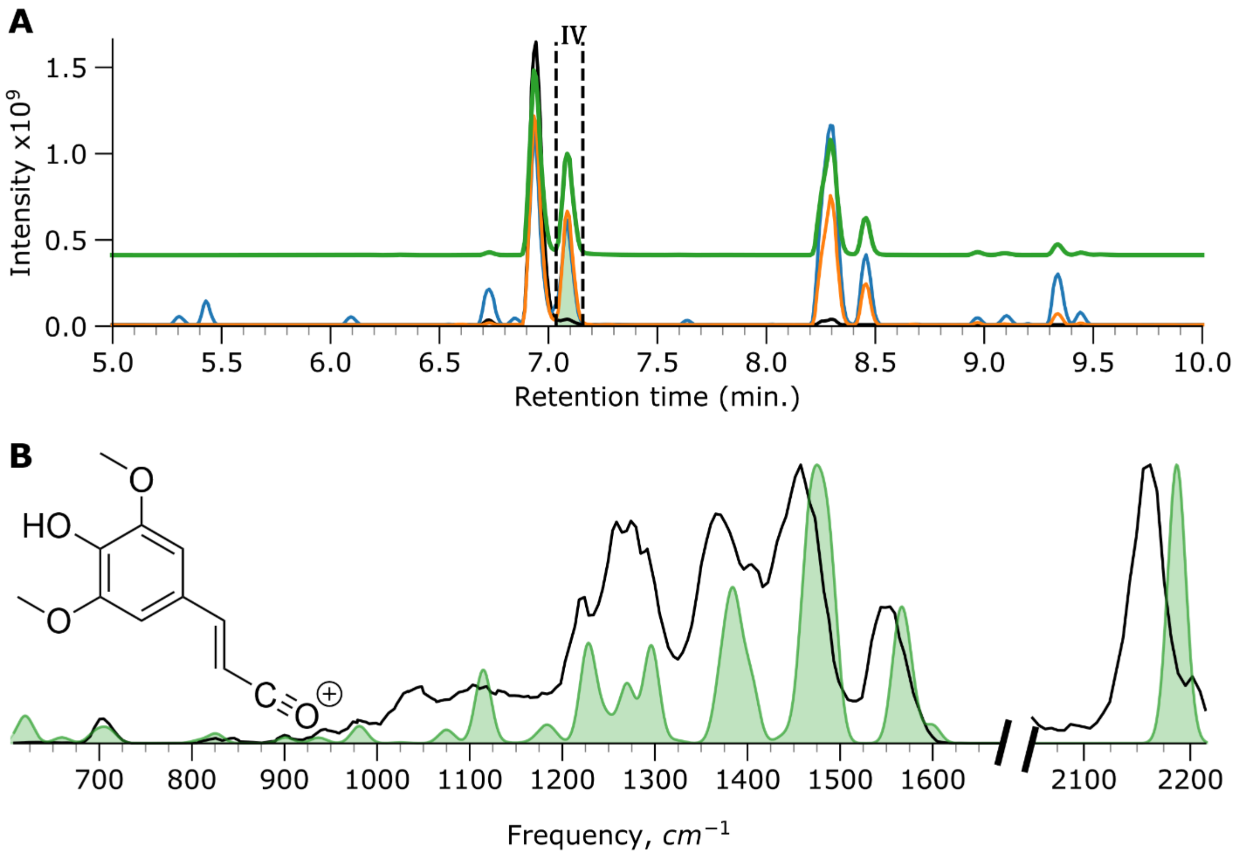

Figure SI 9| Spectrum of the *m/z* 207 ion fractioned from chromatographic feature IV. ***A***: *BPC chromatogram of SM (black trace), SM irradiated by simulated solar radiation (orange trace with vertical dashed lines and blue filled curve indicating LC fractioning), SM irradiated by UV (blue trace), and a normalized extracted ion chromatogram (EIC) curve of the m/z 363 ion (green trace).* ***B***: *Measured IRIS spectrum depicted in black with the computed Boltzmann-weighted average spectrum of all conformers for the ester cleaved acylium fragment of trans-SM given as a green filled curve.*
